# Draft Genome Sequence of *Thermaerobacter* sp. Strain PB12/4term, a Thermophilic Facultative Anaerobic Bacterium from Bottom Sediments of Lake Baikal, Russia

**DOI:** 10.1128/MRA.01178-18

**Published:** 2018-11-21

**Authors:** Olga A. Baturina, Olga N. Pavlova, Angelina S. Novikova, Marsel R. Kabilov, Tamara I. Zemskaya

**Affiliations:** a SB RAS Genomics Core Facility, Institute of Chemical Biology and Fundamental Medicine, Siberian Branch of the Russian Academy of Sciences, Novosibirsk, Russia; b Limnological Institute, Siberian Branch of the Russian Academy of Sciences, Irkutsk, Russia; Queens College

## Abstract

Here, we report the draft genome sequence of Thermaerobacter sp. strain PB12/4term, a thermophilic facultative anaerobic bacterium from the bottom sediments of Lake Baikal, Russia, associated with the discharge zone of gas-bearing fluids.

## ANNOUNCEMENT

The genus Thermaerobacter belongs to the *Clostridiales* family XVII, *incertae sedis*, of the order *Clostridiales*, class *Clostridia*, and the phylum *Firmicutes*. This genus has five validly published species (www.bacterio.net/thermaerobacter.html). Thermaerobacter marianensis Takai, Inoue, and Horikoshi 1999 7p75aT is a typical strain isolated from the bottom sediments of the deepest point of the Mariana Trench, Challenger Deep, at a depth of 10,897 m (1). Thermaerobacter species have been described as strict aerobes, having a strictly respiratory type of metabolism with oxygen as the terminal electron acceptor (2). Thermaerobacter species are thermophilic, with growth occurring between 50 and 80°C, with optimum growth between 70 and 75°C (2). They are neutrophiles or alkalophiles, with growth occurring between pH 5 and 10, with optimum pH between 7 and 8.5 (2). They may utilize organic substrates, such as yeast extract, peptone, cellulose, starch, chitin, casein, Casamino Acids, and a variety of sugars, carboxylic acids, and amino acids (2). Their cells are Gram negative or Gram variable using Gram staining (2).

Strain PB12/4term was isolated from the enrichment cultures of the low-temperature surface bottom sediments sampled near the methane seep Posolsk Bank (southern Baikal, Russia). For the initial enrichment, basal medium was used (3) supplemented with 1.5 g/liter NaHCO_3_ and 0.5 g/liter Na_2_S ·9H_2_O at 60°C under a H_2_-CO_2_ (80:20 by volume) gas mixture. The strain PB12/4term was isolated from the highest positive dilution after three rounds of serial dilutions under the basal medium described above.

Phylogenetic analysis was carried out with the MEGA 5.2 software package (4) using neighbor joining and Kimura’s two-parameter algorithm. The branching order was determined by bootstrap analysis of 100 alternative trees. Based on 16S sequence similarity, the organism was found to be a member of the genus Thermaerobacter, with the closest neighbors deemed to be T. nagasakiensis Ts1a and T. subterraneus C21^T^ (99% similarity). Despite the high genetic identity, the strain showed significant physiological and biochemical differences from the closely related members of the genus Thermaerobacter. Compared with other strains of Thermaerobacter, this strain grew under both oxic and anoxic conditions.

To isolate DNA for genome sequencing, biomass of Thermaerobacter sp. PB12/4term was grown on the basal medium described above at 60°C. Genomic DNA was isolated from cultured cells by using a DNeasy PowerSoil kit (Qiagen) according to the manufacturer’s instructions. Then, genomic DNA was sheared in a microTUBE AFA fiber snap-cap tube using a Covaris S2 instrument. The DNA library with an average size of insert of about 600 bp was prepared using a NEBNext Ultra II DNA library prep kit for Illumina (NEB) and dual-index NEBNext multiplex oligos (NEB). Whole-genome sequencing of the Thermaerobacter sp. PB12/4term library was conducted with reagent kit version 3 (600-cycle) on a MiSeq genome sequencer (Illumina) at the SB RAS Genomics Core Facility (ICBFM SB RAS, Novosibirsk, Russia). The entire genome was assembled *de novo* with SPAdes version 3.12 (5), gap closure and scaffolding were performed by SSPACE version 3.0 (6) (-x 1 -m 15 -o 3 -n 10 -v 0 -g 0 -T 12 -S 0 and other parameters by default) and GapFiller version 1.10 (7) (-m 20 -d 100 -T 12 -i 2 and other parameters by default), and contig reordering was done using the Mauve Aligner version 2.4.0 (8). The automatic functional annotation results were obtained using the NCBI Prokaryotic Genome Annotation Pipeline (9).

A total of 2,097,054 reads were assembled to a draft genome of 2,796,303 nucleotides (nt) at 150-fold coverage with 72.2% GC content. The genome sequence consists of 35 contigs with an *N*_50_ value of 155,884 nt. The PB12/4term strain genome contains 2,344 protein-coding genes, 2 rRNA operons, 43 tRNA genes, 103 pseudogenes, and 1 transfer-mRNA (tmRNA).

### Data availability.

This whole-genome shotgun project has been deposited in GenBank under the accession number QUWU00000000. Raw sequencing data sets have been registered in the NCBI SRA database under the accession number SRP166065. The 16S rRNA gene sequences were deposited in GenBank under the accession number KY492688.
